# Reversible Crystallization of Argatroban after Subcutaneous Application in Pigs

**DOI:** 10.1155/2012/560513

**Published:** 2012-09-03

**Authors:** Mercedes Lopez, Goetz Nowak

**Affiliations:** ^1^Laboratorio de Hemostasia y Genetica Vascular, Centro de Bioquimica y Biofisica, Instituto Venezolano de Investigaciones Cientificas (IVIC), Caracas 1020, Venezuela; ^2^University Hospital Jena, Friedrich Schiller University Jena, Erlanger Allee 101, 07747 Jena, Germany

## Abstract

Argatroban is a thrombin inhibitor used as anticoagulant in patients with heparin-induced thrombocytopenia. It is usually administered as an intravenous bolus followed by infusion. Nevertheless, its pharmacokinetics after subcutaneous administration is unknown. The aim of this study was to assess the pharmacokinetics of two different formulations of argatroban in pigs after subcutaneous administration. Antithrombotic activity in plasma was determined by ecarin chromogenic assay. To visualize the formation of crystals, argatroban was administered to rats into the subcutaneous tissue exposed after removing the skin, and the injection site was photographed at different times. After subcutaneous administration of a sorbitol/ethanol formulation of argatroban in pigs was observed a slow absorption phase was followed by long-lasting levels of this inhibitor. *C*_max_ and AUC_(0−24)_ showed dose-dependent increases, while elimination half-life and *t*_max_ value did not change significantly with dose. In contrast, saline-dissolved argatroban showed a faster absorption phase followed by a shorter elimination half-life. Argatroban dissolved in sorbitol/ethanol leads to long-lasting plasma levels due to the formation and permanent dissolution of a crystalline depot at the injection place. This represents a simple way to deliver argatroban continuously over an extended period which can be beneficial for prophylaxis or treatment of chronic coagulations disorders.

## 1. Introduction

Unfractionated heparin and low molecular weight heparin have been widely used to prevent thrombosis in several patient populations [1–3]. Nevertheless, direct thrombin inhibitors offer several advantages over heparins; they inhibit both free and fibrin-bound thrombin; they are not associated with induction of heparin-induced thrombocytopenia (HIT), and they do not require cofactors such as antithrombin III to exert their effects. For this reason, they could be applied in patients with AT deficiency [4, 5].

Four parenteral direct thrombin inhibitors have been approved for use as anticoagulants in USA; desirudin, lepirudin, bivalirudin, and argatroban. The three first inhibitors are hirudin-derived analogs, while argatroban is a synthetic thrombin inhibitor used in adult patients with heparin-induced thrombocytopenia type II (HIT-II) [4, 6–8].

Argatroban, a derivate from the amino acid L-arginine, was synthesized by Okamoto in Japan [9]. It interacts reversibly with the catalytic site of thrombin with a Ki of 39 nM and possesses a molecular mass of 526.6 Da [10, 11]. The pharmacokinetic profile of argatroban after intravenous administration in healthy volunteers is described by a two-compartment model with first-order elimination. This compound is metabolized mainly by the liver and has a short elimination half-life ranging between 39 and 51 minutes [12–14]. Because of its short half-life, argatroban is usually administered as an intravenous bolus followed by infusion. Argatroban is sparingly soluble in water (about 1 mg·mL^−1^), when administered to a patient, the commercial available  formulation of this inhibitor containing sorbitol and ethanol is diluted to a concentration of 1 mg·mL^−1^ in the form of a mere aqueous solution. The recommended initial dose of argatroban for prophylaxis or treatment of HIT is 2 *μ*g·kg^−1^·min^−1^, titrated to achieve an activated partial thromboplastin time 1.5–3 times the baseline [6, 12, 15]. After initiation of infusion, anticoagulant effects are immediately produced and steady-state levels are maintained until the infusion is discontinued or the dose is adjusted. Upon cessation of infusion, argatroban concentrations rapidly decline remaining little or no argatroban in plasma four hours thereafter [10].

The pharmacokinetics of argatroban after subcutaneous bolus has not been investigated in detail. The aim of this study was to assess the pharmacokinetics of two different formulations of argatroban after subcutaneous administration in pigs, the commercially available sorbitol/ethanol formulation (Novastan), and an aqueous solution with a similar composition to that applied by infusion. 

We have demonstrated that it is possible to achieve long-lasting plasma argatroban levels after subcutaneous administration of this thrombin inhibitor, when it is dissolved in sorbitol/ethanol due to the formation and permanent dissolution of a crystalline depot at the injection place. This could be beneficial for prophylaxis or treatment of chronic coagulation disorders.

## 2. Materials and Methods

### 2.1. Materials

 Argatroban (Novastan) was purchased by Penn Clinical Studies Supply Unit (Gwent, UK). Dry argatroban was supplied by Mitsubishi Pharma (Tokyo, Japan). ECA-T Kits were purchased by Stago (Asnieres, France). Ecarin was purchased from Pentapharm AG (Basel, Switzerland). Pentobarbital-Sodium was acquired from Merial (Hallbergmoos, Germany) and Ethylurethan from Fluka (Taufkirchen, Germany). Human plasma pooled from 10 healthy donors was obtained from Red Cross Centre (Gera, Germany).

### 2.2. Treatments and Blood Sampling

German village pigs (9.8 to 16 kg of body mass) of either sex were initially anaesthetized by an intravenous injection of 20 mg·kg^−1^ pentobarbital-sodium. A catheter (Tygon, 3 mm diameter) was placed in the left jugular vein for blood sampling and then, the pig was tracheotomised for long-time spontaneous respiration. To maintain anaesthesia throughout the experiment, ethylurethane was given intraperitoneally in single doses of 0.5 g·kg^−1^ every 2-3 h. All experiments were performed in accordance with the guidelines for animal experimentation approved by the Regierungspraesidium, Thuringia, Germany.

In order to investigate the influence of argatroban vehicle on its pharmacokinetic profile, two different formulations were used. The commercially available argatroban solution (Novastan) contains, per mL, 100 mg argatroban, 750 mg D-sorbitol, and 1000 mg dehydrated ethanol. The other formulation was prepared dissolving argatroban in an acidic saline solution to a final concentration of 1 mg·mL^−1^. The pH of this solution was adjusted to pH 7.0.

Furthermore, to assess the influence of the volume of injection on the pharmacokinetics of the sorbitol/ethanol formulation, it was diluted keeping the same proportion of sorbitol and ethanol as in the original ampoule.

Argatroban was administered behind the ear as a single subcutaneous bolus of 0.5 or 2 mg·kg^−1^ of body weight. The blood samples were drawn into plastic tubes containing 3.13% sodium citrate before dosing and at definite time intervals thereafter. Then, blood samples were centrifuged (1000 g for 10 min) to obtain citrated plasma. Urine samples were also collected using a bladder catheter and frozen at −20°C until further analysis.

At the end of the experiment, the animals were sacrificed and the injection site of argatroban was removed and prepared for macroscopic analysis in order to determine argatroban-induced local toxic effects. Furthermore, systemic effects as bleeding were investigated by examination of all major tissues and organs.

### 2.3. Antithrombin Activity

Argatroban concentrations were determined in plasma and urine samples by ecarin clotting time (ECT) as previously described [16]. Furthermore, antithrombin activity in plasma samples was also determined by ecarin chromogenic assay (ECA) [17].

### 2.4. In Vivo Microcrystals Formation

 Adult male wistar rats (approximately 250 g) were anaesthetized with 2 g·kg^−1^ ethylurethane. Each argatroban formulation or their respective vehicles were administered into the subcutaneous tissue exposed after removing the skin. Then, the injection site was photographed to defined time intervals with a digital camera under 10x magnification in a Nikon stereo microscope.

### 2.5. Pharmacokinetic Calculations

 The pharmacokinetic parameters were calculated as follows: peak concentration (*C *_max_) and time to reach it (*t *_max_) were taken directly from the data. The area under the curve from 0 to 24 h (AUC_0−24_) was calculated using the trapezoidal method. The *β*-phase half life considered as elimination half life (*t*_1/2*β*_) was calculated from the slope of the terminal portion of the log plasma argatroban concentration versus time curve [18].

### 2.6. Statistical Analysis

It was performed using JMP7 software (SAS Institute, Cary, NC, USA), and the nonparametric Wilcoxon Mann-Whitney test was used to determine significance between groups. Significance was defined as a *P *value of less than 0.05.

## 3. Results

**Figure 1:**
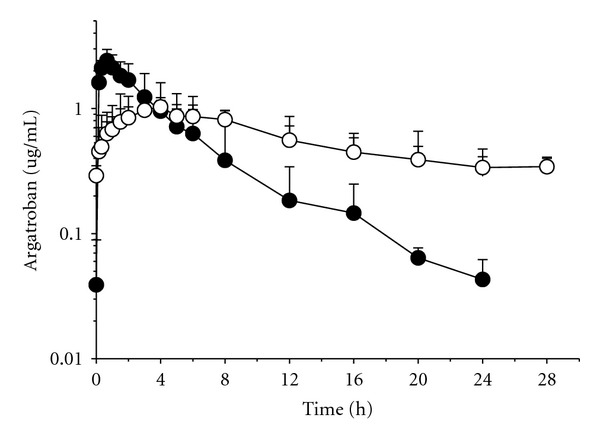
Plasma concentrations in pigs after s.c. administration of 0.5 mg·kg^−1^ of argatroban in two different formulations: sorbitol/ethanol (open circles) and saline solution (closed circles). Results are expressed as the mean plasma concentration (*μ*g·mL^−1^) of *n* = 5, showing the standard deviation at each sampling time.

The time course of plasma concentrations of argatroban in pigs after s.c. administration of two different formulations as determined by ECA is shown in Figure 1.

Subcutaneous administered saline-dissolved argatroban has an immediate effect on plasma argatroban concentrations, a *C*_max⁡_ of 2.48 ± 0.57 *μ*g·mL^−1^ was reached at a *t*_max⁡_ of 0.6 ± 0.28 h which is an evidence of a faster absorption of this argatroban preparation in comparison to the sorbitol/ethanol formulation that showed a *C*_max⁡_ value of 1.072 ± 0.13 *μ*g·mL^−1^ with a *t*_max⁡_ of 3.80 ± 1.30 h. The elimination of argatroban was also steeper when it was dissolved in saline solution showing a half-life significantly shorter than that of the sorbitol/ethanol formulation (4.38 ± 0.43 h versus 14.46 ± 3.91 h; *P* < 0.05). Comparison of the pharmacokinetic parameters between both argatroban formulations at the same dose (0.5 mg·kg^−1^) is shown in Table 1. 

**Figure 2:**
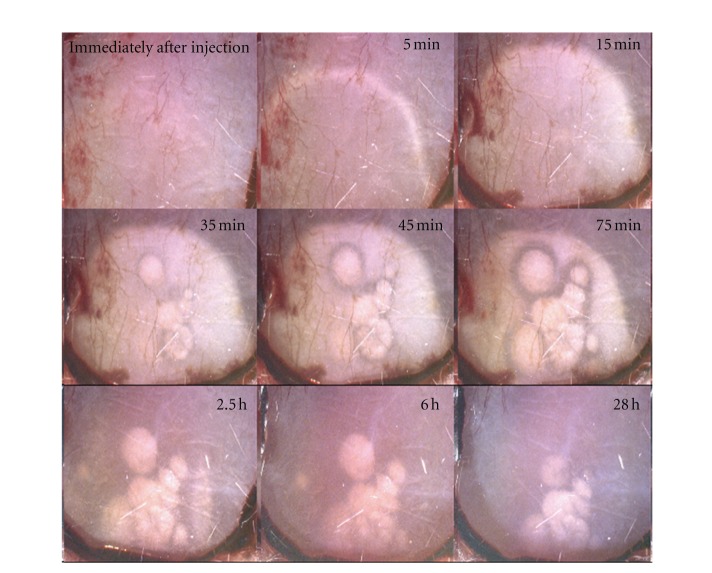
A total of 1.2 mg of argatroban in 40 *μ*L of sorbitol/ethanol mixture was injected subcutaneously into an adult rat. The pictures represent the images with 10x magnification.

The slow absorption and elimination phases observed upon injection of the sorbitol/ethanol formulation led us to hypothesize that argatroban crystallizes after being subcutaneously injected. To formally confirm this hypothesis, we have administered both argatroban formulations as a single subcutaneous bolus in rats and the injection site was photographed at different time intervals.  Figure 2 shows argatroban crystals under 10x magnification, which are visible 35 minutes after application of the sorbitol/ethanol formulation (i.e., 1.25 mg/40 *μ*L at one site/rat). The crystal depot grew up to 75 min and thereafter, it was dissolved slowly being visible even 28 hours upon injection. Crystal formation was not observed after subcutaneous administration of the acidic saline formulation (data not shown).

**Figure 3:**
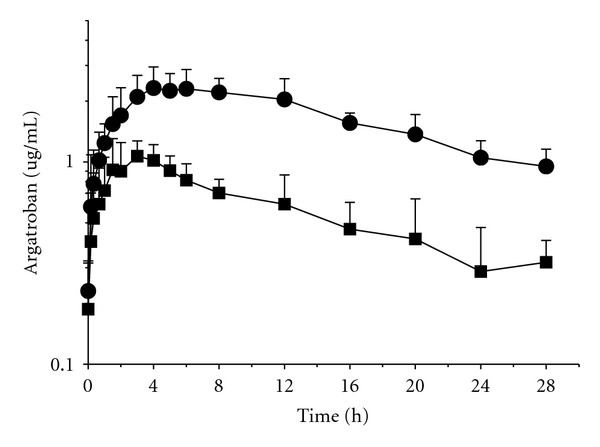
Time course of the argatroban plasma concentration after s.c. administration of 0.5 mg·kg^−1^ (closed squares) or 2 mg·kg^−1^ (closed circles) of sorbitol/ethanol formulation. Results are expressed as the mean plasma concentration (*μ*g·mL^−1^) of *n* = 5, showing the standard deviation at each sampling time.

We have also investigated the kinetics after subcutaneous administration of two different doses of the sorbitol/ethanol formulation (Figure 3). After 0.5 mg·kg^−1^ dose, a *C*_max⁡_ of 1.07 ± 0.13 *μ*g·mL^−1^ was reached at a *t*_max⁡_ of 3.8 ± 1.3 h; while after 2 mg·kg^−1^ dose, a *C*_max⁡_ of 2.59 ± 0.42 *μ*g·mL^−1^ was observed at a *t*_max⁡_ of 4.25 ± 0.5 h. Thereafter, argatroban plasma levels decreased slowly with a half-life ranging between 14.46 and 16.46 hours. Argatroban plasma levels of about 0.3 and 1 *μ*g·mL^−1^ were observed one day after subcutaneous administration of 0.5 and 2 mg·kg^−1^, respectively. A summary of the pharmacokinetic parameters after subcutaneous application of two different dose of the sorbitol/ethanol formulation is displayed in Table 2. *C*_max⁡_ and AUC_(0−24)_ showed significant dose-dependent increases while elimination half-life and *t*_max⁡_ value did not change significantly with dose.

**Figure 4:**
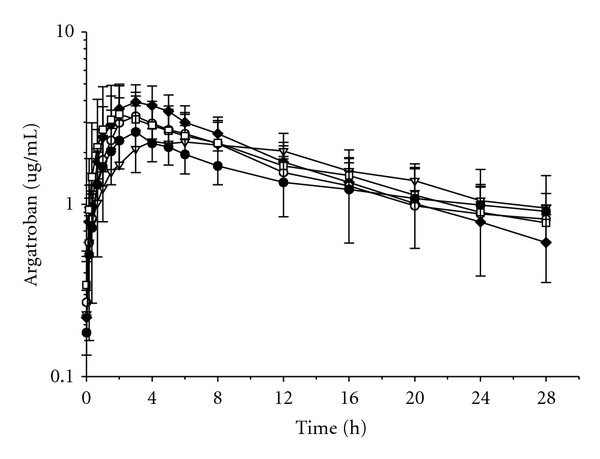
Plasma argatroban concentrations after s.c. administration of 2 mg·kg^−1^ in pigs using solutions with different concentrations: 5 (open triangles), 15 (closed rhombus), 30 (open circles), 50 (open squares), and 100 mg·mL^−1^ (closed circles). Results are expressed as the mean plasma concentration (*μ*g·mL^−1^) of *n* = 5, showing the standard deviation at each sampling time.

Subcutaneous pharmacokinetic profile of argatroban dissolved in sorbitol/ethanol did not change due to variations in the volume of injection. As shown in Figure 4, time course curves of this inhibitor were identical by injecting solutions with different argatroban concentrations (5, 15, 30, 50, and 100 mg·mL^−1^) to reach a dose of 2 mg·kg^−1^ body weight.

Local bleeding, toxic reactions, and other side effects were not observed in all animals used in this study. Moreover, injection sites were rarely identifiable one day after argatroban administration.

As expected for a drug metabolized mainly by the liver, analysis of the cumulative urine excretion of argatroban showed that only about 15–20% of the administered antithrombotic active amount was recovered in urine. This result was similar for both administered argatroban formulations (data not shown).

## 4. Discussion and Conclusions

Argatroban infusions are suitable only for a short-term acute treatment but are not recommended for treatment of chronic coagulation disorders. In this study, we have assessed a new route of administration in order to achieve a longer duration of its biological activity. Indeed, we have investigated the pharmacokinetic of two different formulations of argatroban after subcutaneous administration in pigs, the commercially available sorbitol/ethanol formulation and an aqueous solution with similar composition to that applied by infusion.

The pharmacokinetic profiles of both argatroban formulations were dramatically different as determined by ECA. Indeed, the aqueous formulation has a quicker onset of effect (as evidenced by lower *t*_max⁡_ with higher *C*_max⁡_ value) but with a concomitant reduced duration of action due to a lower *t*_1/2*β*_ value (4.4 hours) in comparison with the sorbitol/ethanol preparation (14.46 hours).

The variation in the pharmacokinetic behavior of both argatroban formulations led us to propose the hypothesis that after subcutaneous injection of the sorbitol/ethanol formulation, argatroban crystallizes forming a depot that dissolves slowly in the interstitial fluid before absorption into microvasculature of the subcutaneous tissue. Thus, the intrinsic solubility of the crystal depot will govern the pharmacokinetics of argatroban and explain the slow absorption and elimination phases observed after administration of the sorbitol/ethanol formulation prolonging the duration of the antithrombotic effect of this inhibitor.

To confirm experimentally this hypothesis, both argatroban formulations were applied to rats into the subcutaneous tissue exposed after removing the skin and the precipitation/dissolution process was followed optically. In this way, we have confirmed that a microcrystalline depot is formed few minutes after injection of the sorbitol/ethanol formulation followed by a long-time dissolution process which correlates with the long-lasting biological effect of argatroban after subcutaneous application of this formulation.

Indeed, the elimination half-life of the sorbitol/ethanol formulation is longer than that of other anticoagulants commonly used in patients such as low molecular weight heparins (LMWHs) (*t*_1/2*β*_ between 3 to 6 h after s.c. injection); which in turn is longer than that of unfractionated heparins (UFH) [19, 20]. Likewise, the half-life of the direct thrombin inhibitor hirudin is about 60 min when administered intravenously and 120 min after subcutaneous administration, which can ensure stable plasma concentrations and antithrombotic activity for a period of approximately 12 hours [21, 22]. Only PEG-hirudin, which was generated by conjugation of hirudin with two molecules of polyethylene glycol (PEG)-5000, possesses significantly longer duration of action than nonconjugated hirudin permitting once daily subcutaneous administration [23, 24]. This period of time is similar to the duration of the action of a subcutaneous bolus of the sorbitol/ethanol formulation of argatroban.

One of the oldest and simplest approaches employed to prolonging the time action of therapeutic protein is to inject it in a form of a suspension of amorphous or microcrystalline particle that dissolves slowly in the interstitial fluid before absorption into microvasculature of the subcutaneous or intramuscular tissue [25–27]. In this preclinical study, we have observed crystallization only after subcutaneous administration of argatroban dissolved in sorbitol/ethanol. It might be possible that argatroban becomes crystallized at the injection site due to the fast absorption of ethanol. This phenomenon can represent an easy and attractive way to deliver this inhibitor continuously over an extended period to maintain the argatroban levels within the therapeutic window without the limitations of the commonly used infusion.

Furthermore, the formation of almost constant levels of argatroban within the therapeutic or prophylactic window as a result of its crystallization at the injection site, its dose-proportional effect, and predictable anticoagulant behaviour might decrease the appearance of side-effects, in comparison with other anticoagulants, such as dabigatran or rivaroxaban, two orally available inhibitors of thrombin and FXa, respectively, whose levels might change considerably reaching peak plasma concentrations in toxic range or dropping under the desired range.

Moreover, the slow dissolution process of argatroban crystals followed by its immediate distribution does not lead to local substance accumulation and the occurrence of unwanted side effects as local bleeding or cell toxic effects. Only a mechanical irritation could be induced by the crystals; however, the amount of substance deposited under the skin would be relatively small and it will be surrounded from connective tissue, lowering the probability of occurrence of this complication.

We did not find any alteration on the pharmacokinetic profile of the sorbitol/ethanol formulation by variations of up to 20 times in the volume of injection to reach the same argatroban dose which indicates that the volume of injection does not influence the formation of the crystalline depot and the release kinetics of argatroban from this drug reservoir.

On the other hand, dose increments of argatroban led to higher *C*_max⁡_ and AUC_(0−24)_ values due to the formation of a bigger crystalline depot, nevertheless, other parameters such as elimination half-life and *t*_max⁡_ value have not changed significantly with dose.

Currently, the most commonly used test for monitoring argatroban therapy is the activated partial thromboplastin time (aPTT). However, aPTT assay becomes less responsive at increasing concentrations of thrombin inhibitors; therefore, toxic blood levels cannot be detected or prevented by measuring aPTT. Further limitations are the dependence of aPTT on the reagent used and the high interindividual variations of the clotting time values [28]. Likewise, argatroban has a dose-dependent effect on prothrombin time (PT) but unfortunately also on INR values. For this reason, the results obtained by PT might change depending on the used reagent which limits the reliability of this test [29]. Furthermore, ecarin clotting time (ECT) reaction time is strongly correlated with plasma argatroban concentrations. This test is considered as a highly sensitive and precise method for determination of thrombin inhibitors over a wide concentration range with low interindividual variations, but it is affected by low levels of prothrombin and fibrinogen [16]. The Ecarin Chromogenic Assay (ECA) overcomes these drawbacks making it a more reliable and accurate test for direct quantification of argatroban concentrations [17]. In this study, we have determined antithrombin activity not only by ECA but also by ECT. However, the results were similar by both methods. In this paper, we only showed the results obtained by ECA, because this method represents an advanced version than ECT.

In summary, the special subcutaneous argatroban pharmacokinetics is caused by its immediate crystallization which might be due to a faster absorption of the ethanol present in the vehicle. This crystal depot acts as a drug reservoir which releases argatroban continuously at a rate determined in a large extent by its solubility into the interstitial fluids leading to the sustained absorption of the drug into the microvasculature. This long duration of the biological effect after subcutaneous application of argatroban might be very beneficial for treatment of chronic diseases, thus avoiding the need of infusion, repetitive dosing, or surgery for implanting depots.

Crystallization after subcutaneous injection could be useful to prolong the biological efficiency of other slightly water-soluble compounds.

## Figures and Tables

**Table 1:** Pharmacokinetic parameters of argatroban in pigs after s.c. administration of 0.5 mg·kg^−1^ of two different formulations of this inhibitor: sorbitol/ethanol and saline solution. Values are the mean of *n* = 5, showing the standard deviation. *P < 0.05; **P < 0.01.

	Argatroban formulation	
	Sorbitol/ethanol	Saline solution
*t*_1/2β_ (h)	14.46 ± 3.91	4.38 ± 0.43*
*C*_max⁡_ (*μ*g·mL^−1^)	1.072 ± 0.13	2.48 ± 0.57**
*t*_max⁡_ (h)	3.80 ± 1.30	0.60 ± 0.28**
AUC_0–24_ (*μ*g·mL^−1^·h)	14.34 ± 2.42	9.47 ± 1.35*

*t*_1/2β_: elimination half-life; *C*_max⁡_: maximum concentration to *t*_max⁡_; *t*_max⁡_: time of maximum concentration; AUC_0–24_: area under the concentration-time curve from 0 to 24 hours.

**Table 2:** Pharmacokinetic parameters of argatroban in pigs after single subcutaneous doses of 0.5 or 2 mg·kg^−1^ of the sorbitol/ethanol formulation. Values are the mean of *n* = 5, showing the standard deviation. *P < 0.05; **P < 0.01.

	Argatroban dose	
	0.5 mg·kg^−1^	2 mg·kg^−1^
*t*_1/2β_ (h)	14.46 ± 3.91	16.46 ± 3.96
*C*_max⁡_ (*μ*g*·*mL^−1^)	1.07 ± 0.13	2.59 ± 0.42*
*t*_max⁡_ (h)	3.80 ± 1.30	4.25 ± 0.50
AUC_0–24_ (*μ*g·mL^−1^·h)	14.34 ± 2.42	40.76 ± 5.12**

*t*_1/2β_: elimination half-life; *C*_max⁡_: maximum concentration to *t*_max⁡_; *t*_max⁡_: time of maximum concentration; AUC_0–24_: area under the concentration-time curve from 0 to 24 hours.
